# The association between language-based task-functional magnetic resonance imaging hemodynamics and baseline GABA+ and glutamate–glutamine measured in pre-supplementary motor area: A pilot study in an aging model

**DOI:** 10.3389/fpsyt.2022.904845

**Published:** 2022-08-15

**Authors:** Lisa C. Krishnamurthy, Isabella Paredes Spir, Natalie O. Rocha, Brian J. Soher, Edward J. Auerbach, Bruce A. Crosson, Venkatagiri Krishnamurthy

**Affiliations:** ^1^Center for Visual and Neurocognitive Rehabilitation, Atlanta VA Healthcare System, Decatur, GA, United States; ^2^Department of Physics & Astronomy, Georgia State University, Atlanta, GA, United States; ^3^Department of Radiology and Imaging Sciences, Emory University, Atlanta, GA, United States; ^4^Department of Biology, Georgia State University, Atlanta, GA, United States; ^5^Center for Advanced MR Development, Department of Radiology, Duke University, Durham, NC, United States; ^6^Brain Imaging and Analysis Center, Duke University, Durham, NC, United States; ^7^Department of Radiology, University of Minnesota, Minneapolis, MN, United States; ^8^Center for Magnetic Resonance Research, University of Minnesota, Minneapolis, MN, United States; ^9^Department of Psychology, Georgia State University, Atlanta, GA, United States; ^10^Department of Neurology, Emory University, Atlanta, GA, United States; ^11^Division of Geriatrics and Gerontology, Department of Medicine, Emory University, Atlanta, GA, United States

**Keywords:** GABA, glutamate, fMRI, language, MRS, aging, pre-supplementary motor area, hemodynamics

## Abstract

Aging is a natural phenomenon that elicits slow and progressive cerebrovascular and neurophysiological changes that eventually lead to cognitive decline. The objective of this pilot study is to examine the association of GABA+ and glutamate–glutamine (Glx) complex with language-based blood oxygen level dependent (BOLD) hemodynamics in an aging model. More specifically, using standard BOLD we will first attempt to validate whether previously reported findings for BOLD amplitude and resting neurochemical relationships hold in an aging model. Secondly, we will investigate how our recently established neurosensitized task-BOLD energetics relate to resting GABA+ and Glx, especially accounting for titration of task difficulty. To support the above endeavors, we optimize the baseline fitting for edited magnetic resonance spectroscopy (MRS) difference spectra to sensitize GABA+ and Glx concentrations to aging-related differences. We identify a spline-knot spacing of 0.6ppm to yield the optimal aging-related differences in GABA+ and Glx. The optimized MRS values were then graduated to relate to task-BOLD hemodynamics. Our results did not replicate previous findings that relate task-BOLD amplitude and resting GABA+ and Glx. However, we did identify neurochemistry relationships with the vascularly-driven dispersion component of the hemodynamic response function, specifically in older participants. In terms of neuro-sensitized BOLD energetics and the underlying role of GABA+ and Glx, our data suggests that the task demands are supported by both neurometabolites depending on the difficulty of the task stimuli. Another novelty is that we developed task-based functional parcellation of pre-SMA using both groups. In sum, we are the first to demonstrate that multimodal task-fMRI and MRS studies are beneficial to improve our understanding of the aging brain physiology, and to set the platform to better inform approaches for clinical care in aging-related neurovascular diseases. We also urge future studies to replicate our findings in a larger population incorporating a lifespan framework.

## Introduction

Aging is a biological process that encompasses a multitude of physical changes, including loss of brain volume and tissue atrophy (1–3), plausibly in response to underlying cerebrovascular and neurophysiological changes (4). However, the process of how aging-related physical and physiological changes ultimately lead to cognitive decline is still unclear. In the quest to unravel the multi-level complexities of brain function, the rise of neuroimaging techniques has dramatically changed the scene over the last three decades. Specifically, multi-modal neuroimaging approaches that combine magnetic resonance spectroscopy (MRS) and functional magnetic resonance imaging (fMRI) are highly informative in such endeavors (for a descriptive review see (5) and a comprehensive meta-analysis see (6)), but all reports to date examine younger or midlife populations. While we recently showed that the flow-metabolism coupling (i.e., associations between neurometabolites and cerebral blood flow) is critical in aging-related cognitive decline (7), the role of neurometabolites in task engagement is yet to be explored in an aging model, Furthermore, most reports relate MRS measures with task-BOLD amplitude, but the shape of the hemodynamic response is also of great importance when relating to neurochemistry (8), especially in an aging model.

It is well established that human brain function is governed by the release of neurotransmitters such as the brain’s major inhibitory neurotransmitter, gamma-aminobutyric acid (GABA), and the brain’s major excitatory neurotransmitter, glutamate (9). Proton (^1^H) MRS studies indicate an aging-related decline in GABA+ and glutamate–glutamine (Glx) concentrations (10–15) while other reports show no aging-related change in GABA+ or Glx concentration (16, 17). These high-quality reports are landmark articles but combining MRS data with other modalities such as task-fMRI requires advancements in the processing of MRS data to reduce measurement noise and increase detection sensitivity. One source of aging-related variability arises due to differences in the modeled baseline (18) during linear combination modeling, as implemented in LCModel (19, 20). A recent report of baseline-fitting optimization in j-edited MRS to quantify GABA+ in a younger cohort identified a spline-knot of 0.55 ppm as the optimal spacing (21). In the present study, we refine and optimize the baseline modeling to ensure the detection of aging-related differences in brain metabolites. Once the optimal analysis to assess aging-related GABA+ and Glx concentration differences is determined, we further combine the MRS data with task-fMRI BOLD hemodynamics.

Our group recently showed that correcting blood oxygen level-dependent (BOLD) task-fMRI signal for baseline cerebral blood flow (CBF) enhances the specificity and sensitivity of task-fMRI results in both younger and older participants (4). Our correction approach also enhanced the correlation between BOLD energetics and in-scanner behavioral performance, indicating that the task-fMRI BOLD signal was more sensitized to its neural components (which we henceforth term as “neurosensitized” task-fMRI BOLD signal). A deeper glimpse into aging-related differences in such neurosensitized task-induced BOLD energetics revealed – (i) compared to young adults, the older participants showed a delayed language-related task activity possibly due to compromised vascular compliance, and (ii) functional evolution of neurosensitized BOLD activity revealed biphasic BOLD dynamics in both groups where greater BOLD deactivation suggested greater semantic demand or increased premium on domain general executive functioning in response to task difficulty (4).

The brain’s excitatory and inhibitory neurotransmitters likely influence the downstream metabolically-driven BOLD hemodynamics and energetics (22), but more experimental evidence across the lifespan to advance our knowledge in this aspect is needed. A majority of reports combining baseline-MRS and task-fMRI describe an inverse relationship between GABA and standard (i.e., not neurosensitized, or unsensitized) task-BOLD amplitude (6, 23). A handful of other groups reported a positive relationship between the brain’s major excitatory neurotransmitter, glutamate, and task-BOLD amplitude (6). Of note is that most combined MRS-fMRI reports only address the relationship between GABA+ and BOLD amplitude, but do not quantify the hemodynamics to assess how the neurotransmitter concentration relates to the shape of the hemodynamics. To our knowledge, there is only one report that showed a positive relationship between GABA+ concentration and task-BOLD latency (time-to-peak) and dispersion (full-width half-maximum) (8) but is limited to a younger cohort. However, because we know that our previously reported task-fMRI has differences in latency and shape between younger and older cohorts (4), it is imperative to investigate how baseline-MRS measures might correlate to these aging-related changes.

Thus, the overarching objective of this pilot study is to combine task-fMRI and baseline-MRS to investigate the interplay of GABA+ and Glx with task-BOLD hemodynamics in an aging model. The specific goals are as follows: (1) to optimize the baseline fitting of j-edited difference MR spectra to improve sensitivity to aging-related changes in GABA+ and Glx and reduce quantification errors to detect multimodal relationships, (2) to investigate whether previously reported relationships between GABA+ and Glx concentration and language task-BOLD hemodynamics hold in an aging model, and (3) explore the relationships of GABA+ and Glx with neurosensitized BOLD energetics that are parsed as a function of evolving task difficulty in repetitive within-category member generation. We chose to collect MRS from the pre-supplementary motor area (pre-SMA) for this study. While aging-related changes in GABA+ and Glx have previously been reported in pre-SMA (10), recent language-based research articles have reported multiple potential functions of pre-SMA in language production and processing (specifically, verbal fluency) (24–28). In summary, the study outcomes for the above goals are reported for baseline-MRS and task-fMRI measurements from the pre-SMA brain area in a younger and older cohort.

## Materials and methods

### General procedures

Forty-three participants were recruited *via* community flyers or volunteer registry. Participants were included in the study if between 18 and 34 or 60 and 89 years of age, right-handed, native English speaker, and without a history of depression or neurological disease. Participants were excluded if hospitalized within the past 6 months, presented with significant cognitive impairments defined as a Montreal Cognitive Assessment (MoCA) score of <24, or have any magnetic resonance imaging (MRI) contraindications, including implanted devices or severe claustrophobia. All participants provided informed consent in a process that was approved by the Emory University Institutional Review Board and Atlanta VA Research Oversight committee. All consent procedures followed the Declaration of Helsinki.

Each participant completed two study sessions: a cognitive testing session and an MRI session. After quality control, 28 participants remained with good MR spectra, defined as a Creatine linewidth full width half maximum (FWHM) of <18 Hz. A subset of 19 participants with MRS data was identified to also have task-fMRI data that met quality control standards for motion. The data is reported on the 28 and 19 participants, depending on the data type. The participant demographics for the cohort of 28 and 19 are reported in Table 1.

**TABLE 1:** Participant demographics for the cohort of *N* = 28 and the diminished cohort of *N* = 19.

	Cohort of 28 subjects		Cohort of 19 subjects	
		
	Younger (*N* = 14)	Older (*N* = 14)	Younger (*N* = 9)	Older (*N* = 10)
Age	23.71 ± 3.65	68.38 ± 5.85	23.22 ± 3.15	67.90 ± 4.77
Sex	6 females	6 females	3 females	4 females
Years of education	15.50 ± 1.51	15.93 ± 0.83	15.56 ± 1.51	15.90 ± 0.99
MOCA	28.00 ± 1.52	27.29 ± 1.68	27.89 ± 1.69	27.60 ± 1.65

All MR imaging and spectroscopy data were collected on a Siemens 3T Prisma with radio frequency (RF) transmission achieved *via* the body coil and RF reception achieved with a 64 channel phased array head coil. The participants were made comfortable with foam padding placed around the head and instructed not to move. The participants were presented with a white crosshair on black background for all scans except for the task-fMRI. The high-resolution T1w MPRAGE, task functional MRI (task-fMRI), and pseudo continuous arterial spin labeling (pCASL) MRI were presented in our previous study (4). In the same session, MR spectroscopy (MRS) data was collected that was sensitized to assess localized GABA+ and glutamate concentrations. In this report we optimize the MRS analysis and combine the information with fMRI.

### GABA+ MRS acquisition and pre-processing

The J-edited (29) MRS acquisition utilized the Center for Magnetic Resonance Research (CMRR) Spectroscopy Tools Mescher–Garwood Point Resolved Spectroscopy (MEGA-PRESS) (30) sequence to separate the small GABA+ signals from the rest of the MR spectrum (TR = 2,000 ms, TE = 68 ms, voxel size = 3 cm × 3 cm × 3 cm, acquisition bandwidth = 2,000 Hz, acquisition duration = 1,024 ms, vector size = 2,048, VAPOR water suppression bandwidth = 135 Hz, editing pulse bandwidth = 53 Hz, ON editing pulse = 1.9 ppm, OFF editing pulse = 7.5 ppm, total scan duration = 10 min). Each free induction decay (FID) was collected and stored separately for use in preprocessing. The CMRR Spectroscopy Tools FAST(EST)MAP (31, 32) was used to achieve a high-quality shim in the pre-SMA. The voxel was centered in midline pre-SMA on a high resolution T1w MPRAGE (sagittal 3D acquisition, TR = 2,530 ms, TE = 2.96 ms, TI = 1,100 ms, FA = 7 deg, FOV = 256 mm × 240 mm, 176 slices, voxel size = 1 mm × 1 mm × 1 mm, partial Fourier = 7/8, acquisition bandwidth = 130 Hz/px, total scan duration = 8:53 min) by trained study personnel as described in Supplementary Section 1 “GABA planning in pre supplementary motor area (preSMA).” The pre-SMA was chosen for this experimental paradigm for two reasons: (1) previous studies have shown aging-related changes in this area (10), and (2) the pre-SMA is a domain-general language area (27) that the accompanying task-fMRI is designed to engage. An unsuppressed water (H_2_O) spectrum with matching acquisition parameters was also collected from the same region, except that the TR = 10 s to allow for full T_1_ relaxation.

The raw FIDs were imported into Matlab for pre-processing using in-house routines. Specifically, the FIDs were (1) corrected for phase and frequency drift (33) to improve the linewidth of the averaged spectra, (2) removal of spectra with frequency drifts greater than 10 Hz that generally occurs due to motion, (3) alignment of ON and OFF spectra at 3 ppm to reduce the occurrence of subtraction artifacts in the difference spectra, followed by (4) subtraction of averaged ON and OFF spectra. The complex time-domain data was output in .RAW format for further quantification in LCModel (19, 20) as described in the next section. After LCmodel quantification, the water scaled metabolite concentration was cerebrospinal fluid (CSF) corrected (34) based on voxel registrations and tissue segmentations performed in Gannet (35). The parsed voxel was also transformed into MNI space *via* the warp matrix computed from the high-resolution T1w image for overlap across participants and localization of pre-SMA task-BOLD data.

### Optimization of LCModel baseline fitting on GABA+ MRS difference spectra

Simulated basis sets were generated using the VESPA (VErsatile Simulation, Pulses, and Analysis) (36, 37) package. The RF pulses used for the CMRR MEGA-PRESS sequence on the Siemens Prisma VE11C Syngo platform were imported into the Vespa-Pulse application and then used in the Vespa-Simulation application to simulate the full MEGA-PRESS data acquisition for both ON and OFF editing pulses. The ON/OFF spectra were then post-processed to create DIFF spectra and results output for use in the LCmodel fitting software. The following metabolites were simulated in Vespa-Simulation MEGA-PRESS: Alanine, Aspartate, Creatine, Phosphocreatine, GABA, Glucose, Glutamine, Glutamate, Glycerophosphorylcholine, Glutathione, Myo-inositol, Lactate, *N*-acetyl aspartate, *N*-acetyl aspartate glutamate, Scyllo-inositol, Taurine, Choline, and Glycine. The LCModel options specified for this project in the Control Parameter file are detailed in Supplementary Section 2 “LCModel control parameter settings.”

Once imported into LCModel, the DIFF spectra were modeled using the VESPA simulated basis sets in addition to a modeled baseline. The stiffness of the baseline is controlled by assigning a number to the dkntmn option in the Control Parameter file. We tested 6 different baselines: dkntmn = 0.2, 0.4, 0.6, 0.8, 1.0, and no baseline (noBline). The baseline with a smaller fractional number such as 0.2 has more flexibility, while larger numbers such as 1.0 indicate a stiffer baseline. We also tested the model fitting without baseline (noBline) because the LCModel user manual suggests turning off baseline fitting for modeling MEGA-PRESS difference spectra. The metabolite SNR was computed by exporting the LCModel output *via* the lcoord command into text format and imported into matlab for further processing. The model fit (including baseline), and residuals of the LCModel computation are used together to determine the metabolite SNR in Equation 1:


(1) $$S\,N\,R_{m\,e\,t\,a\,b\,o\,l\,i\,t\,e}^{k}=\frac{\max\left(\left|F\,i\,t_{m\,e\,t\,a\,b\,o\,l\,i\,t\,e}^{k}-F\,i\,t_{b\,a\,s\,e\,l\,i\,n\,e}^{k}\right|\right)}{2\cdot r\,m\,s\,\left(r\,e\,s\,i\,d\,u\,a\,l\,s_{k}\right)}$$

Where SNR^k^_metabolite_ is the signal to noise ratio of either GABA or Glx for the kth dkntmn spline knot spacing, Fit^k^_metabolite_ is the fitted GABA or Glutamate+glutamine curve for the kth dkntmn spline knot spacing, Fit^k^_baseline_ is the fitted baseline curve for the kth dkntmn spline knot spacing, max(| |) indicates the maximum absolute value of the difference between metabolite and baseline fits, residuals_*k*_ is the LCModel output residual between the input spectra and fitted model for the kth dkntmn spline knot spacing, and rms indicates to compute the root mean square. The optimal baseline fitting was assessed based on GABA+ and Glx signal to noise ratio (SNR) as well as goodness of fit, as determined by LCmodel’s Cramer-Rao lower bounds (CRLB). The goal was to determine the optimal baseline to use during the MRS analysis and then promote the GABA+ and Glx results to combine with task-fMRI data.

### Language task-fMRI acquisition

Three runs of a sparse-sampled (38) blood oxygenation level dependent (BOLD) functional MRI (fMRI) were collected during overt word generation to assess language-related task activation. The MR sequence parameters of the multiband single-shot gradient recalled echo-planar imaging (EPI) sequence were as follows: FoV = 220 mm × 220 mm, multiband acceleration factor = 6, matrix = 100 × 100, 72 slices, interleaved axial acquisition, slice thickness = 2 mm, repetition time (TR) = 4,000 ms (1,000 ms image acquisition + 3,000 ms delay during which participants were cued to make an overt response), echo time (TE) = 33 ms, acquisition bandwidth = 2,500 Hz/px, flip angle (FA) = 90°, and 78 measurements per run. EPI geometric distortions were corrected using a pair of spin-echo EPI scans with opposite phase encoding directions (“top-up”) (39).

**FIGURE 1:**
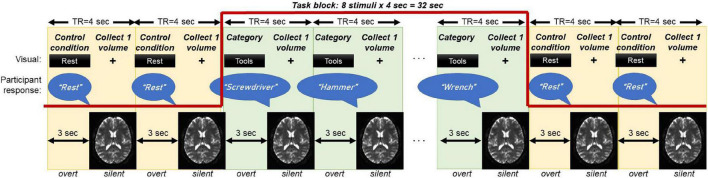
Schematic of the overt category member generation (i.e., semantic fluency) task that has been optimized to fit a sparse-sampled MRI acquisition. The participant is visually presented a category for 3 s, during which they generate a novel exemplar of that category. During the category presentation, no MRI data is collected (effectively a TR gap). During the MRI data collection, the screen switches to a “+” fixation cross for 1 s, during which the participant is silent and still to reduce the influence of motion. One block contains 8 stimuli (32 s). To control for activation from speech, the task block is flanked by presentations of the word “Rest,” during which the participant also says the word “Rest.” Three runs of 6 blocks are collected, for a total of 3 × 6 × 8 = 144 stimuli.

The overt category member generation task is graphically displayed in Figure 1. A category (e.g., “tools”) was visually displayed in print. Upon reading the category, the participant was asked to generate an exemplar word (e.g., “screwdriver”) associated with that category within 3 s of the stimulus presentation. Following the display of the category, the screen switched to a “+” fixation cross-hair during the 1 s EPI acquisition of a single BOLD volume, during which the participants was instructed to remain silent and still. A block contained 8 presentations of the same category, to which the participant was instructed to provide one novel exemplar each time. Each task run consisted of six blocks with different categories followed by jittered blocks of 3–5 TRs where the participant was instructed to read the word “rest” aloud. The rest blocks were designed to provide a contrast between semantic engagement and motor speech production. A total of 3 task runs were collected on each participant, such that a total of 3 × 6 = 18 categories (144 stimuli) were collected. All responses were recorded using an MR compatible microphone (OptoAcoustics Inc., Israel) affixed to the head coil. Participants were trained on this task immediately prior to scanning and instructed to generate the word “pass” if unable to generate an exemplar within 3 sec.

### pCASL MRI data acquisition and cerebral blood flow quantification

The pCASL MRI acquisition and CBF quantification is described in our previous article (4). Briefly, a 2D pCASL EPI sequence was used to assess regional brain perfusion. The images were collected at the magnet isocenter to obtain an adequate blood label. The sequence parameters are as follows: FOV = 220 mm × 220 mm, matrix = 64 × 64, TR = 4,080 ms, TE = 13 ms, GRAPPA factor = 2, twenty 5 mm axial slices in ascending order with a 1 mm gap, post-labeling delay (PLD) = 1.8 s, labeling time = 1,500 ms, 47 pairs of label and control acquisitions with a total scan time of 6 min 36 s. The participants were instructed to stay awake with their eyes open and be still during the duration of the scan. A fully relaxed proton density-weighted scan (M0) with similar parameters except for TR = 10 s with 2 averages were acquired to convert the perfusion signal to absolute CBF value.

The pCASL time-course was corrected for bulk-head motion and pairs of volumes were censored if the head motion was greater than 0.5 mm. The pCASL data was then spatially smoothed (Gaussian kernel FWHM = 6 mm), followed by pairwise subtraction of control and label images which were averaged to generate the mean perfusion image. The signal was converted to CBF in physiological units (mL/100g/min) by dividing the perfusion image with the smoothed M0 image and applying a single-compartment model (40). The CBF maps were spatially transformed into MNI space and resampled to the task-fMRI matrix size in preparation for the neuro-sensitization step described next.

### Processing and neuro-sensitization of task-fMRI

Neuro-sensitization is a method developed by our group to remove the influence of baseline CBF on task-fMRI data at the group level (4). Quality control (QC) of the unprocessed task fMRI time series was carried out using FBIRN (41), and only those data sets that passed QC criteria for task-fMRI [described in Supplementary Section 1 in (4)] were promoted for further analysis. Briefly, the task-fMRI datasets were corrected for slice timing, bulk-head motion, and EPI distortion, and then spatially transformed to MNI space, smoothed using a 4 mm FWHM Gaussian kernel, and scaled to obtain task-induced relative % BOLD change. A 48-s hemodynamic response function (HRF) was extracted using deconvolution (AFNI’s 3dDeconvolve). To neuro-sensitize the task-fMRI, the area under the curve (AUC) of the HRF was computed, z-transformed, and then regressed against the baseline CBF at the group level. The neuro-sensitization process removes physiological variability and has been shown to relate better with behavior (4).

### Extraction of task-fMRI BOLD amplitude, time-to-peak, and full width half maximum from whole MRS voxel

The average HRF was computed from each participant’s MRS voxel, confined to the grey matter (GM). The GM ribbon was identified by segmenting the T1w high-resolution anatomical scan with FSL’s fast, spatially transformed to MNI space, and then resampled to task-fMRI matrix size. After spatially averaging, the HRF with a 4 s temporal resolution was imported into Matlab, and spline interpolated with the function spline() to a temporal resolution of 0.5 s. The resulting spline interpolated HRF has a high enough temporal sampling to extract the FWHM. The peak amplitudes of the original HRF and spline-interpolated HRF were spot-checked to make sure the interpolation did not distort the HRF shape. In effect, each participant had a single HRF curve with a temporal resolution of 0.5 s representative of the pre-SMA location.

Based on our previous work with this dataset (4), we expected the HRF to have a biphasic response. Thus, to identify the maximum “peak” of the HRF during the stimulus block, regardless of positive or negative activation, the absolute value of the interpolated HRF was fed into Matlab’s findpeaks() function. The sign of the peak was subsequently applied. The findpeaks() function also extracts the time-to-peak (ttp) and FWHM. The HRF metrics of amplitude, time-to-peak, and FWHM were extracted for every participant and further used in statistical analysis to relate with neurochemistry.

### Functional parcellation of MRS voxel using neuro-sensitized task-fMRI

Based on our previous work (4), behaviorally, the semantic fluency task was more difficult toward the end of the task block, especially for more difficult semantic categories. Therefore, we were able to divide the HRF into three segments: segment 1 was acquired during generation of the first 4 exemplars, segment 2, during generation of the last 4 exemplars, and segment 3 was a 16 s post-task period during which the post-stimulus undershoot (PSU) was expected to develop. Thus, by dividing the HRF into 3 segments, the dynamic evolution of the BOLD signal can be assessed. More specifically, the first segment was defined for the first 16 s (Seg1 = first 4 words), the second segment from 17th through 32 s (Seg2 = last 4 words), and 3rd segment was defined from 33rd to 48th seconds for post-stimulus BOLD activity (Seg3 = PSU). On each participant, the voxel-wise AUC for a given segment of the HRF was estimated and then z-transformed for subsequent secondary group analyses.

A 3dttest was applied to the segment-wise Z(AUC) to identify areas of significant task-activity within the confines of the MRS voxel overlap. The younger and older segment-wise Z(AUC) maps were separately tested against zero and voxel-wise thresholded at *p* ≤ 0.01 and FWE corrected for multiple-comparison correction at a false positive rate ≤ 5%. Significant clusters for segments 1, 2, and 3 were binarized to extract average Z(AUC) from each participant for further statistical analysis with neurochemistry.

### Statistical tests relating neurochemistry to task- fMRI metrics

It is expected that both inhibitory and excitatory neurotransmitters will relate to BOLD hemodynamics and neuroenergetics (22). Thus, we applied a multiple linear regression (MLR) model to test how much GABA+ (inhibitory), Glx (excitatory), participant age, and the cross-term GABA*Glx (inhibitory–excitatory interaction) may describe HRF metrics of BOLD amplitude, time-to-peak, and FWHM, as well as neuro-sensitized and unsensitized BOLD energetics Z(AUC).

Seven models were tested to describe HRF metrics with GABA, Glx, and Age. Equations 2–8 show the construction of each model and the name of the model referred to in the text.


(2) $$\text{Glx}-\text{only}\;B\,O\,L\,D_{j}^{i}=A+B\cdot G\,l\,x+e$$

(3) $$\mathrm{GABA}-\text{only}\;B\,O\,L\,D_{j}^{i}=A+B\cdot G\,A\,B\,A+e$$

(4) $$\text{GABA}-\text{by}-\text{Glx}\;B\,O\,L\,D_{j}^{i}=A+B\cdot G\,l\,x+C\cdot G\,A\,B\,A$$

+D⋅(G⁢A⁢B⁢A⋅G⁢l⁢x)+e

(5) $$\mathrm{Age}-\text{only}\;B\,O\,L\,D_{j}^{i}=A+B\cdot A\,g\,e+e$$

(6) $$\text{Age}-\text{by}-\text{Glx}\;B\,O\,L\,D_{j}^{i}=A+B\cdot G\,l\,x+C\cdot A\,g\,e$$

+D⋅(A⁢g⁢e⋅G⁢l⁢x)+e

(7) $$\text{Age}-\text{by}-\text{GABA}\;B\,O\,L\,D_{j}^{i}=A+B\cdot G\,A\,B\,A+C\cdot A\,g\,e$$

+D⋅(A⁢g⁢e⋅G⁢A⁢B⁢A)+e

(8) $$\text{Age}-\text{by}-\text{Glx}-\text{by}-\mathrm{GABA}\,B\,O\,L\,D_{j}^{i}=A+B\cdot G\,l\,x+C\cdot G\,A\,B\,A$$

+D⋅A⁢g⁢e+E⋅(G⁢A⁢B⁢A⋅G⁢l⁢x)

+F⋅(G⁢A⁢B⁢A⋅A⁢g⁢e)+G⋅(G⁢l⁢x⋅A⁢g⁢e)+e

where i is the BOLD HRF metric [i.e., amplitude or FWHM or ttp or Z(AUC)], *j* denotes the ROI or whole MRS voxel, GABA is the optimized water scaled CSF-corrected GABA+ concentration, Glx is the optimized water scaled CSF corrected Glx concentration, Age is the participant’s age, and A, B, C, D, E, F, and G are parameters that are fit for during the modeling, and *e* is the error term. All linear and multiple linear regression modeling was accomplished in JMP Pro16 using Standard Least Squares fit. The results of the whole model fit are reported with F and associated *p*-value. The results of each parameter fit are reported with *t* and associated *p*-value. Consistent with fMRI statistical thresholding, we chose a relatively stringent threshold of *p* ≤ 0.01 for MLR interpretation. Further, for segment-wise pre-SMA parcellation analysis, if we obtained more than one ROI for a given segment, then we conducted a Bonferroni correction for multiple-comparisons.

## Results

### MRS and task- fMRI data from younger and older participants

We inspected the quality of our MR spectra for both younger and older participants and noted a FWHM linewidth at the 3 ppm moiety of 7.8 ± 2.4 Hz and 10.0 ± 3.6 Hz, respectively. The older participants have a trending higher creatine linewidth (*t* = −1.87, *p* = 0.08), likely due to field inhomogeneities induced by brain atrophy.

**FIGURE 2:**
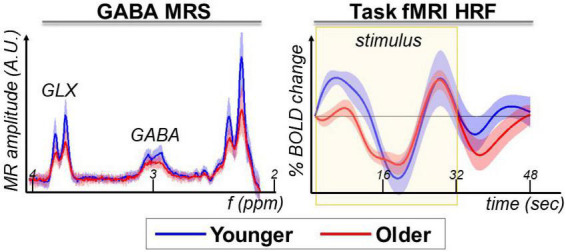
GABA+ MRS and task-fMRI are the magnetic resonance modalities used in this study to assess aging-related differences.

Visual inspection of the difference spectra at the Glx and GABA+ resonances indicates a lower MRS amplitude for older participants (Figure 2, GABA+ MRS panel). Considering this observation, we will optimize the MRS analysis to detect such aging-related differences (described below in the next section).

The task-fMRI HRF extracted from the pre-SMA voxel area shows the expected biphasic response during the stimulus task block, with greater differences between younger and older at the beginning of the task block (Figure 2, task-fMRI HRF panel). In this study, we assessed the relationship between GABA+ MRS and language task-fMRI within the pre-SMA. For a more detailed whole-brain report on the task-fMRI results from this cohort, please see our previous publication (4).

### LCModel baseline fitting on GABA+ MRS difference spectra

The pre-processed difference spectra were imported into LCModel to fit for brain metabolites including GABA+ and Glx, while modulating the fitted baseline stiffness to optimize the fit for aging-related differences. Because the raw spectra (Figure 2) show a difference between younger and older participants, we expect to quantify a difference in the fitted metabolites. This expectation is further solidified by the LCModel fitting of “no baseline,” which provides a significant difference in GABA+ (*t* = 2.71, *p* = 0.01) and Glx (*t* = 5.08, *p* < 0.0001).

**FIGURE 3:**
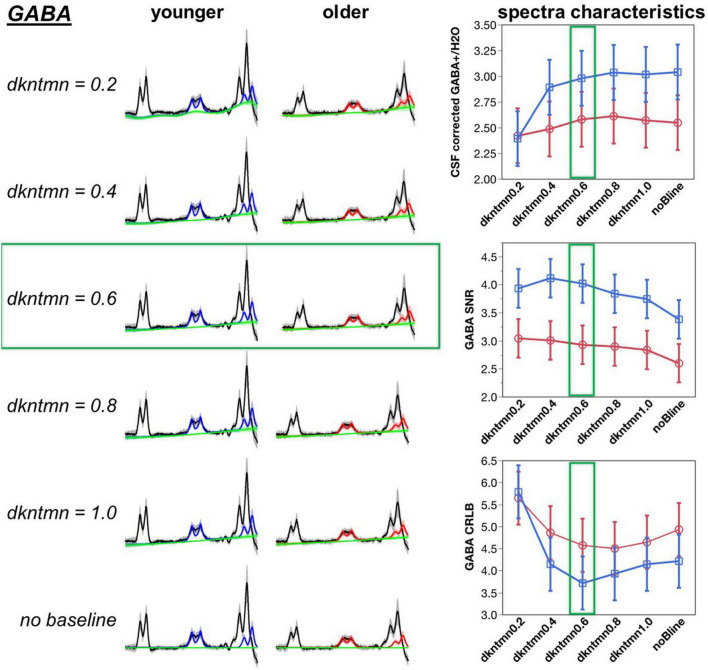
Optimization of difference signal baseline fitting for GABA. A lower dkntmn indicates a more flexible baseline and higher dkntmn indicates a stiffer baseline. Fitting results for the GABA+ moiety for dkntmn 0.2–1.0 ppm and no baseline are shown for younger and older cohorts. Black line = average raw spectra, blue = GABA fit for younger, red = GABA fit for older, green line = baseline.

**FIGURE 4:**
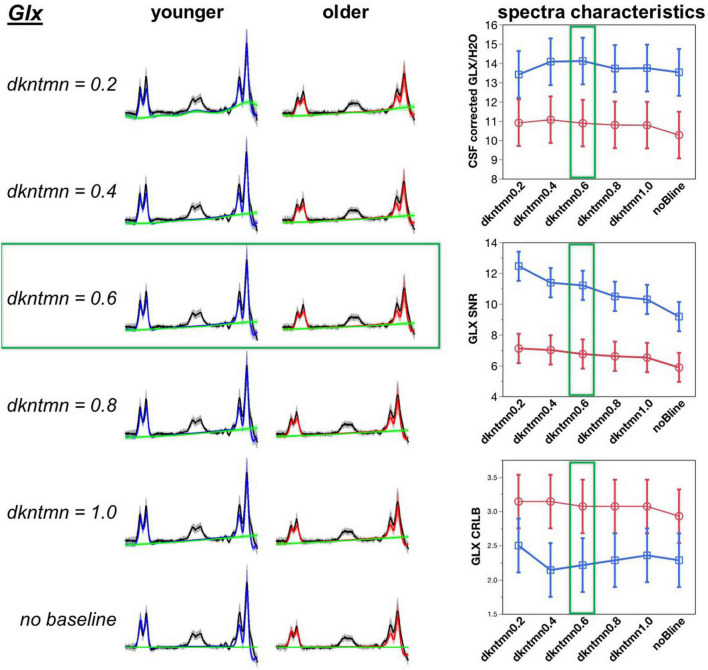
Optimization of difference signal baseline fitting for Glx. A lower dkntmn indicates a more flexible baseline and higher dkntmn indicates a stiffer baseline. Fitting results for the Glx moiety for dkntmn 0.2–1.0 ppm and no baseline are shown for younger and older cohorts. Black line = average raw spectra, blue = GABA fit for younger, red = GABA fit for older, green line = baseline.

Next, we tested if the GABA+ and Glx fit could be improved by adding a baseline during the fitting process (Figures 3, 4). The baseline accounts for experimental conditions that are not described by the basis set – such as shimming issues, chemical shift artifacts, and underlying macromolecules and lipids that are not included in the basis set. In LCModel, the flexibility of the baseline can be controlled using the control parameter “dkntmn.” A lower dkntmn indicates a more flexible baseline, while a higher dkntmn prescribes a stiffer baseline. We chose to test spline-knot spacings dkntmn = 0.2, 0.4, 0.6, 0.8, and 1.0 ppm, as well as no baseline (noBline) fitting and compare the output concentration, metabolite signal to noise ratio (SNR), and the quality of the fit quantified by Cramer-Rao lower bounds (CRLB).

A baseline will theoretically account for more deviations in the model, thereby reducing the fitting residuals and increasing the SNR of the metabolites. Using a comparison with the overall average, the noBline fitting of GABA+ resulted in a significantly lower SNR (*t* = −2.73, *p* = 0.007). Thus, adding a baseline during the modeling of a difference fit can significantly improve the amount of described signal. Using a Student’s *t*-test pairwise comparison, we observed a dkntmn = 0.2 (*t* = 2.36, *p* = 0.02), dkntmn = 0.4 (*t* = 2.70, *p* = 0.008), and dkntmn = 0.6 (*t* = 2.29, *p* = 0.02) with a significantly higher GABA+ SNR than the noBline fitting. All other pairs were not significantly different in terms of GABA+ SNR. Thus, from SNR itself, we identified that a relatively flexible baseline (dkntmn = 0.2, 0.4, or 0.6) can improve metabolite SNR. In addition, we attempted to narrow down the choice of baseline by inspecting the quality of fit with CRLB.

The CRLB is an estimate of the variance in the estimator parameter, such that a lower CRLB reflects a good quality estimation of the fitted metabolite. Using a Student’s pairwise comparison-test, we observed the CRLB of dkntmn = 0.2 to be significantly higher than the remaining fits, including the noBline control fit (*t* = 3.75, *p* = 0.0002), suggesting that dkntmn = 0.2 was not the optimal setting. Further, using a comparison with overall average, the dkntmn = 0.2 was again greater (*t* = 5.72, *p* < 0.0001), while dkntmn = 0.6 was significantly lower than the average (*t* = −2.27, *p* = 0.02). Thus, we note that a very flexible baseline such as dkntmn = 0.2 removes important signal attributes on a difference signal. On the other hand, a moderately flexible baseline such as dkntmn = 0.6 provides an improved metabolite SNR while decreasing the CRLB significantly, and thus we confidently moved forward with dkntmn = 0.6 for subsequent steps of MRS analyses. For Glx, the CRLB was not different across different baseline fittings.

### Age-related differences in optimized GABA+ and Glx quantification

**FIGURE 5:**
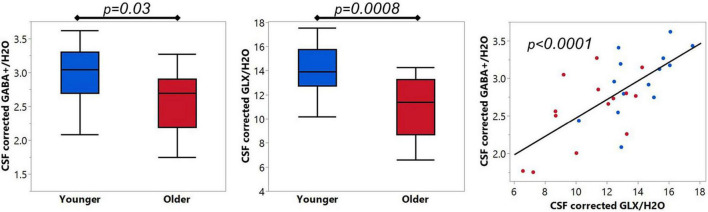
**(Left and Middle)** panels show comparison of optimized GABA+ and Glx between younger (*N* = 14) and older (*N* = 14) cohorts. **(Right)** panel show*s* the expected linear relationship between GABA+ and Glx. Blue = younger, red = older.

Using the optimized baseline stiffness of dkntmn = 0.6, the mean CSF corrected GABA+/H_2_O in pre-SMA was 2.98 ± 0.43 i.u. and 2.58 ± 0.48 i.u. for younger and older, respectively (Figure 5). The CSF corrected GABA+/H_2_O difference between younger and older was significantly different (*t* = 2.32, *p* = 0.03), with Cohen’s *d* effect size = 0.88. Using the optimized baseline stiffness of dkntmn = 0.6, the mean CSF corrected Glx/H_2_O in pre-SMA was 14.11 ± 1.97 i.u. and 10.89 ± 2.50 i.u. for younger and older, respectively (Figure 5). The CSF corrected Glx/H_2_O difference between younger and older was also significantly different (*t* = 3.79, *p* = 0.0008), with Cohen’s *d* effect size = 1.43. As expected, the CSF corrected GABA+/H_2_O and Glx/H_2_O were positively correlated (*R*^2^ = 0.48, *F* = 24.03, *p* < 0.0001, Figure 5).

### Hemodynamic response function metrics from the whole MRS voxel and relationship to neurochemistry

**FIGURE 6:**
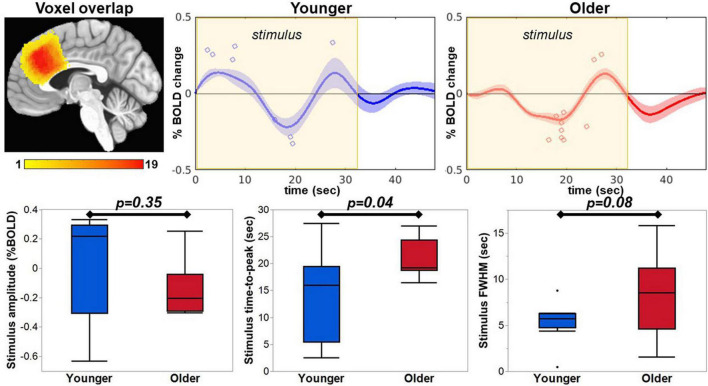
The HRF was extracted from each participant’s MRS voxel location, which were in a similar location covering the pre-SMA as seen in the voxel overlap image. The maximum peak within the stimulus window was extracted and is indicated with a blue circle for the younger group and red circle for the older group on the respective average HRF. The extracted amplitude, time-to-peak, and FWHM are plotted for comparison between the groups.

After optimizing the quantification of GABA+ and Glx, we investigated the relationship between neurochemistry and task-fMRI metrics from the average GM ribbon within the whole MRS voxel. To do so, each participant’s HRF was extracted from their respective MRS voxel location in pre-SMA. The general location of the extracted HRF is similar across participants, as seen by the voxel overlap displayed in Figure 6. The pre-SMA HRF in response to an overt member-generation language task was biphasic in nature, with an initial increase, followed by a decrease in the BOLD signal. Visually, the initial increase was stronger in the younger participants compared to the older (Figure 6, top row). The maximum HRF peak within the stimulus block was automatically detected (blue and red circles on HRF plots, Figure 6) to extract metrics of amplitude, time-to-peak, and FWHM for each participant. Within the stimulus block, the peak BOLD amplitude was not different between younger and older cohorts (*t* = −0.98, *p* = 0.35). However, the timing and duration of the HRF within the stimulus block show that the older cohort had a significantly greater latency (ttp, *t* = 2.29, *p* = 0.04) and a trending wider dispersion of the activation profile (FWHM, *t* = 1.91, *p* = 0.08), especially in the early stages of the task-block (segment 1). The PSU peak had no group differences in amplitude (*t* = −1.30, *p* = 0.21), time-to-peak (*t* = 1.43, *p* = 0.17), or FWHM (*t* = 0.58, *p* = 0.57).

The HRF metrics were then further related to GABA+, Glx and age *via* simple/multiple linear regression using seven statistical models (Equations 2–8). The quality of fit was ranked using Adjusted *R*^2^ as described in Supplementary Section 3 “Identifying a neurophysiological model to describe HRF metrics and Z(AUC).” The comprehensive table of Adjusted *R*^2^ for all participants can be found in Supplementary Table 2. As summarized in Table 2, age-only model best describes the ttp (*p* = 0.02), and GABA*GLX*age model best describes the FWHM (*p* = 0.02) with no individual parameters being significant. Looking at each individual group, the youngers did not have any significant relationship, but older group showed GABA*Glx interaction to influence FWHM (*p* = 0.03) wherein each of those individual parameters also being at or above trending significance (GABA: *p* = 0.02 and Glx: *p* = 0.01). Note that the HRF amplitude did not have any significant relationships at individual group level or all combined. Plots of significant and trending results can be seen in Supplementary Section 4 “Relationships from Tables 2, 3.” Note that for stringent *p* ≤ 0.01 threshold, we defined trending relationships for *p*-value between 0.011 and 0.05.

**TABLE 2:** Best fit neurophysiological model for HRF amplitude, time-to-peak, and FWHM.

	Best fit model	*R* ^2^	Adj *R*^2^	*F*	*p*	Parameter	β	*t*	*p*
**All**									
HRF amplitude	None	–	–	–	–	–	–	–	–
HRF time-to-peak	**Age-only**	**0.29**	**0.24**	**6.79**	0.02*	**Age**	**0.17**	**2.61**	0.02*
HRF FWHM	GABA*Glx*Age	**0.67**	**0.50**	**3.99**	0.02*	GABA	−4.14	−1.91	0.08
						Glx	0.50	0.87	0.40
						Age	0.07	1.49	0.16
						GABA*Glx	0.10	0.15	0.88
						GABA*Age	−0.20	−1.76	0.10
						Glx*Age	0.04	1.70	0.12
**Younger**									
HRF Amplitude	None	–	–	–	–	–	–	–	–
HRF time-to-peak	None	–	–	–	–	–	–	–	–
HRF FWHM	None	–	–	–	–	–	–	–	–
**Older**									
HRF Amplitude	None	–	–	–	–	–	–	–	–
HRF time-to-peak	None	–	–	–	–	–	–	–	–
HRF FWHM	GABA*Glx	**0.76**	**0.63**	**6.18**	0.03*	**GABA**	−**7.57**	−**3.03**	0.02*
						**Glx**	1.48	**3.55**	0.01**
						GABA*Glx	0.54	0.73	0.49

β denotes the model coefficient, and F, t, R^2^, Adj. R^2^ and p denote standard statistical parameters for F-statistics, t-statistics, proportion of the variance, proportion of variance adjusted for number of predictors in the model and alpha threshold, respectively.

**significant at p ≤ 0.01, *trending at 0.01 < p < 0.05. Bold values indicate significant or trending relationships.

**TABLE 3:** Best fit ROI-level neurophysiological model for BOLD energetics.

		Best fit model	*R* ^2^	Adj. *R*^2^	*F*	*p*	Parameter	β	*t*	*p*
**ROI1 Z(AUC)**										
Seg1-anterior 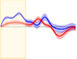	Unsensitized	Age-only	0.28	0.24	6.66	0.02*	Age	−0.02	−2.58	0.02*
	Neurosensitized	None	–	–	–	–	–	–	–	–
**ROI2 Z(AUC)**										
Seg1-mid 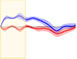	Unsensitized	**Age-only**	**0.61**	**0.59**	**27.10**	<0.0001**	**Age**	−**0.03**	−**5.21**	<0.0001**
	Neurosensitized	**Age × GABA**	**0.63**	**0.56**	**8.56**	0.002**	**Age**	−**0.03**	−**4.21**	0.0008**
							GABA	0.17	0.49	0.63
							Age × GABA	0.02	1.39	0.19
**ROI3 Z(AUC)**										
Seg2-mid 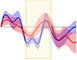	Unsensitized	None	–	–	–	–	–	–	–	–
	Neurosensitized	Glx-only	0.28	0.24	6.68	0.02*	Glx	−0.25	−2.59	0.02*
**ROI4 Z(AUC)**										
Seg3-mid 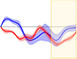	Unsensitized	Age-only	0.23	0.19	5.18	0.04*	Age	−0.03	−2.28	0.04*
	Neurosensitized	Age-only	0.28	0.24	6.73	0.02*	Age	−0.03	−2.59	0.02*
**ROI5 Z(AUC)**										
Seg1-posterior 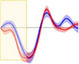	Unsensitized	Age-only	0.22	0.18	4.84	0.04*	Age	−0.02	−2.20	0.04*
	Neurosensitized	Age-only	0.25	0.20	5.64	0.03*	Age	−0.02	−2.37	0.03*

β denotes the model coefficient, and F, t, R^2^, Adj. R^2^, and p denote standard statistical parameters for F-statistics, t-statistics, proportion of the variance, proportion of variance adjusted for number of predictors in the model and alpha threshold, respectively. *ANOVA of model is trending at 0.01 < p < 0.05. **ANOVA of model is significant at p ≥ 0.01. Bold values indicate significant or trending relationships.

### Functional parcellation of the MRS voxel

**FIGURE 7:**
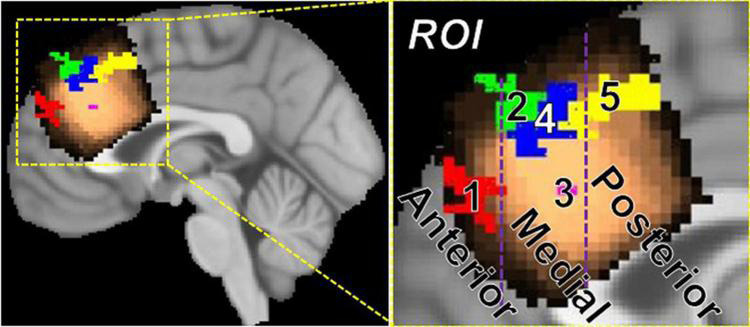
Parcellation of pre-SMA into anterior (*y* > 40 mm), medial (40 mm > *y* > 22 mm), and posterior (*y* < 22 mm) regions using task-fMRI. The anterior pre-SMA contains ROI 1, the mid pre-SMA contains ROI 2, 3, and 4, and the posterior pre-SMA contains ROI 5.

The rationale for functional parcellation was to explore the link between task difficulty and how various sub-regions of pre-SMA (obtained *via* functional parcellation) may play a role in processing stimuli with varying difficulty. Five significant areas of activation (voxel-wise *p* ≤ 0.01, cluster-wise FWE corrected for FPR < 5%) were identified with 3dttest applied to neuro-sensitized Z(AUC) and jointly overlayed on the MRS voxel to functionally parcellate pre-SMA. Figure 7 shows the area of the MRS voxel in copper tone, overlayed by the functional parcellation ROIs in red (ROI1), green (ROI2), magenta (ROI3), blue (ROI4), and yellow (ROI5).

More specifically, by applying a 3dttest to younger participants’ segment 1 Z(AUC), ROI 1 (*x* = 6, *y* = 50.2, *z* = 27.7, cluster = 45) and ROI2 (*x* = 8.1, *y* = 39.3, *z* = 45.1, cluster = 51) were identified. By applying a 3dttest to older participants’ segment 1 Z(AUC), ROI 5 (−3.9,18,47.4, cluster = 84) was identified. By applying a 3dttest to younger participants’ segment 2 Z(AUC), ROI 3 (*x* = 2.2, *y* = 29.3, *z* = 28.0, cluster = 2) was identified. Finally, by applying a 3dttest to older participants’ segment 3 Z(AUC), ROI 4 (*x* = 4, *y* = 33, *z* = 42.7, cluster = 59) was identified. The SMA and pre-SMA are thought to have functional parcellations (27), so we further grouped the resulting ROIs 1–5 into anterior, medial, and posterior regions. Based on these results, we define anterior pre-SMA as *y* > 40 mm, posterior pre-SMA as *y* < 22 mm, and midline pre-SMA as 40 > *y* > 22 mm on the MNI template brain.

### Neuro-sensitzed task-BOLD hemodynamic response function and MRS relationships

As described above, we obtained 3 ROIs (i.e., ROI1, 2, and 5) from segment-1 and thus the associated *p*-value is Bonferroni corrected (=0.01/3 = 0.0033). We obtained only one ROI from segments 2 and 3 and thus no Bonferroni correction was implemented. Finally, for these set of analyses, we defined the trending relationship for *p*-value between 0.0034 and 0.05. We tested seven statistical models (Equations 2–8) to describe the unsensitized and neurosensitized Z(AUC) and ranked the quality of the fit using Adjusted *R*^2^ as described in Supplementary Section 3 “Identifying a neurophysiological model to describe HRF metrics and Z(AUC).” The comprehensive table of Adjusted *R*^2^ for all participants can be found in Supplementary Table 3. Across all participants, the behavioral measures best fit by one of the seven statistical models is summarized in Table 3. At a Bonferroni corrected *p* = 0.0033, ROI2 Z(AUC) is highly significant while the other ROIs show trending relationships, but are reported here in detail to build future hypotheses. Although the whole model reaches significance only in ROI2, specifically in ROI3 through ROI5, neuro-sensitization improves the t-scores of the parameter. In ROI2, while unsensitized showed age-only as the significant parameter, neuro-sensitized Z(AUC) showed significant involvement of age*GABA interaction wherein age was the significant driving parameter. While ROIs 1, 4, and 5 all show trending significance for age-only, neurosensitized ROI3 showed trending significance for Glx. Plots of trending result can be found in Supplementary Section 4 “Relationships from Tables 2, 3.” No trending relationships were identified at the younger or older group level.

## Discussion

Investigating the neurochemical (specifically GABA+ and Glx) underpinnings of BOLD physiology is an important topic in advancing our understanding of brain function as seen through the lens of neuroimaging measurements. No studies of which we are aware have related baseline GABA+ and Glx to task-fMRI in an aging model. In this report, we optimized the MRS baseline fitting to sensitize to aging-related differences. We then further promoted the optimized GABA+ and Glx concentrations for both younger and older cohorts and related to language task-fMRI BOLD hemodynamics of amplitude, latency (i.e., time-to-peak), and dispersion (i.e., FWHM). We also utilized task-difficulty driven segmentation of the BOLD hemodynamics to parcellate the pre-SMA into functional subregions. In both the whole voxel and pre-SMA subregions, we discovered interesting relationships between GABA+, Glx, and task-fMRI BOLD hemodynamics specific to aging-related physiological changes underlying language (i.e., semantic fluency) functions.

### Optimization of magnetic resonance spectroscopy baseline fitting (Goal-1)

Baseline fitting optimization has predominantly been evaluated on unedited MRS data acquisition (19, 20, 42–44). Macromolecules and lipids of unedited MRS contribute to a rapidly varying baseline. Thus, spline-knot spacing of 0.1–0.2 ppm are incorporated into LCModel fitting of unedited MRS spectra (42), but others have argued that such narrow spacing could lead to overfitting (44). On the other hand, a stiff baseline of dkntmn > 1 could lead to bias in the quantified neurochemistry due to underfitting (44). Thus, it is important to optimize the baseline fitting for MRS quantification in LCModel tailored to the sequence and scientific question.

A recent report showed that baseline fitting for MEGA-PRESS difference spectra can be optimized (21). Zoellner et al. tested three spline-knot spacings of 0.25, 0.4, and 0.55, whereas we tested spline-knot spacings of 0.2, 0.4, 0.6, 0.8, and 1.0 and compared to fittings without baseline. There is evidence that the macromolecular and lipid baseline changes across the lifespan (18), thus there was a chance that our study’s baseline optimization may not agree with previously published results because we optimized for aging-related differences. However, our results are in excellent agreement Zoellner at al. because they identified 0.55 ppm as the optimal spline-knot spacing in a younger cohort, and we identified 0.6 ppm as the optimal spacing to sensitize for aging-related differences. Upon testing a spline-knot spacing of 0.2, all aging-related differences were lost, which likely points to an overfitting of the data. On the other hand, applying a baseline to the data (compared to no baseline) improved the SNR and goodness of fit of the data, suggesting that an optimized baseline applied to the model improves metabolite quantification. Although further replication studies are needed to corroborate the choice of 0.55–0.6 ppm spline-knot spacing, the agreement across two separate datasets collected with different MEGA-PRESS implementations and different cohorts increases the confidence in this finding. These optimized GABA+ and Glx concentrations were promoted to relate with task-fMRI hemodynamics.

### GABA+ and Glx relationship with task-induced BOLD hemodynamics: Disagreement with previous reports and new findings (Goal-2)

Early seminal research articles exploring the major neurotransmitter’s relationship with task-fMRI reported an inverse relationship between GABA+ and task-induced BOLD amplitude (8, 45–47), and either a null (45, 46) or positive relationship between glutamate or Glx and task-induced BOLD amplitude (48). In a meta-analysis of MRS and task-fMRI relationships, task BOLD amplitude was related inversely with GABA+ across many brain areas and experimental paradigms (6), as was hypothesized by an early report (8). However, in our current report we find a null relationship between GABA+ and task-BOLD amplitude. The potential reasons for the disagreement with previous reports are multifaceted: (1) our experimental design involved a language task with a biphasic HRF, (2) our measurements were localized to pre-SMA, (3) and our study design involved an aging model. Another experimental factor that may have contributed to the non-corroborating results is the task-fMRI design. While our study incorporated a relatively long (i.e., 32 s) mixed block design, previous reports incorporated shorter blocks or event-related designs. Because the type of task design influences the estimated HRF, perhaps our null relationship between GABA+ and BOLD amplitude are reasonable (and expected) as different tasks require different (and task-appropriate) design. We also did not find a relationship with Glx and task-BOLD amplitude in either younger or older groups, which suggests that Glx-related metabolic demands for a language task in pre-SMA may not be as demanding as compared to visual and affective tasks measured from visual and anterior cingulate cortices. Despite these experimental differences, the lack of a relationship between GABA+ or Glx and task-BOLD amplitude is an important finding, because it signifies that the studies from visual cortex, precuneus, and medial and dorsal prefrontal regions do not generalize across all tasks, BOLD dynamics, brain regions, and age groups. More work is necessary to better understand the influence of neurotransmitter concentration on task-BOLD amplitude.

Typically, most fMRI researchers do not routinely extract deconvolution-based BOLD HRF, but rather just estimate the beta weights from general linear modeling. To our best knowledge, (8) is the only report thus far that has investigated the relationship of neurotransmitters with task-BOLD hemodynamic parameters other than the amplitude. In the present study, we chose to resolve the HRF in younger and older participants and then extract the hemodynamic parameters of amplitude, latency (ttp), and dispersion (FWHM) from the stimulus block. We found that in older participants: (i) the GABA+ concentration was inversely related to the task-BOLD dispersion (FWHM) and (ii) Glx was positively related to the task-BOLD dispersion. This suggests that in older participants, lower resting Glx results in longer delays along with shorter dispersion, and lower resting GABA+ results in wider dispersion of task-induced HRFs. A very important point to note here is that the GABA+ and Glx differentially relates to the dispersion which suggests that the excitation: inhibition balance (EIB) is extremely critical in optimal maintenance of the HRF dispersion. While we must recognize that Glx (which is glutamate–glutamine complex) is not purely excitatory, and that glutamate (Glu) is the precursor for GABA+ synthesis (49), this is a very complex phenomenon that needs further investigation to clearly unravel the role of EIB in regulation of HRF dispersion. The aging-related longer latency observed in BOLD HRF is consistent with our previous work showing delayed task-BOLD activity due to aging-related changes in vascular compliance (4). While our data does not indicate the involvement of GABA+ and/or Glx in aging-related HRF delays, literature suggests that complex molecular signaling involving vasoactive intestinal peptide is critical for the maintenance of glutamate re-uptake (50), and helps GABAergic neurons directly control the cerebral microvasculature (51, 52). Additional work is required to clearly establish the role of GABA+ and glutamate in regulation of vascular function and task-BOLD hemodynamics, particularly in aging and aging-related diseases.

#### Neuro-sensitized task-induced BOLD energetics and its relationship with GABA+ and Glx (Goal-3)

In our previous work involving the same task design in an aging model, we showed that segmenting the task block not only facilitated the neuro-sensitization approach, but also allowed us to bring out subtle aging-related differences as seen *via* functional evolution of BOLD energetics (4). It is important to point out that neuro-sensitization was carried out on the area under the curve (AUC) of each segment, in effect quantifying the BOLD energetics required to carry out the task during a specific segment. The HRF segmentation approach allowed us to examine the relationship of resting GABA+ and Glx with BOLD post-stimulus undershoot (PSU), which is of relevance because the physiological mechanisms underlying BOLD PSU is a highly debated and understudied area within the field (53). Although we found significant changes in PSU in mid pre-SMA after a language task stimulus block, we did not find any significant relationships between GABA+ or Glx and Z(AUC) from segment-3 (i.e., PSU BOLD energetics). As previously suggested by (53), while the origin of BOLD PSU maybe due to sustained increase in metabolic rate of oxygen consumption (CMRO_2_) along with delayed vascular compliance, it is still unclear how much does the metabolic aspects of GABA+ and Glx signaling contribute to PSU-specific CMRO_2_ and delayed vascular compliance.

We carried out a conservative analysis to detect task activation, including voxel-wise thresholding at *p* < 0.01 and FWE to correct for multiple comparison at an FPR < 5%. Further, we were conservative when performing multiple linear regression (MLR) between GABA+, Glx, and task-BOLD AUC by applying Bonferroni correction on a relatively stringent alpha-threshold (*p* < 0.01). Thus, we were thrilled to obtain significant relationships between GABA+, Glx, and task-BOLD AUC in ROI2 (i.e., mid pre-SMA from segment-1). From Table 3 note that the neurosensitized approach provided a superior overall fit for the MLR modeling in mid pre-SMA as compared to the standard or un-sensitized task-BOLD. We did not find any significant relationships between GABA+, Glx and AUC in either younger or older participants, but at the group level it is relevant to note that age-by-GABA interaction is critical in neurosensitized task-BOLD energetics from mid pre-SMA. Further, at the mid pre-SMA level in entire group, Glx was moderately significant in neurosensitized task-BOLD derived from segment-2. Considering that we observed lower GABA+ and Glx in older participants, perhaps mitochondrial degradation (54, 55) due to aging may have disrupted the necessary EIB to support required task demands. Further, in all participants, a lower resting Glx in mid pre-SMA (segment-2) resulted in lower task-BOLD AUC suggesting that task demands are less efficiently processed (i.e., lower AUC) with lower Glx due to age-related compromised regulation of the Glu/Gln cycle (56).

#### Influence of task difficulty on blood oxygen level dependent and GABA+ and Glx relationships (Goal-3)

The proposed within-block segmentation was spurred by intriguing behavioral results (i.e., decrease in accuracy during the second half of the stimulus block) that we previously reported from analyzing the in-scanner task performance (4). For both groups, we noticed that for semantic fluency, generation of words/exemplars for the first four stimuli (i.e., segment-1 = 16 s) was “easier,” while the semantic fluency task became more “difficult” for the last 4 stimuli (i.e., segment-2 = 16 s). Finally, segment-3 of 16 s was the PSU. For the easier segment-1, we note from Table 3 that age-by-GABA interaction inversely relates to task-BOLD AUC. Put together, this suggests that perhaps processing of easier stimuli was efficiently carried out by higher GABA+ and its interaction with age component. As the task becomes more difficult (i.e., segment-2), we note an inverse relationship between Glx and task-BOLD AUC persists suggesting that metabolically more demanding difficult stimuli does require effective involvement of Glx. Considering that an earlier report had noted aging-related decline in glial functioning that supports glutamate–glutamine cycling (55), perhaps efficient metabolic functioning on the glial side is important for processing more difficult task stimuli.

#### Regional heterogeneity of blood oxygen level dependent and GABA+ and Glx relationships within pre-supplementary motor area (Goal-3)

Another novelty that resulted from this study is the functional parcellation of the pre-SMA MRS voxel based on segmented task-BOLD energetics. While SMA is predominantly viewed as a speech motor control brain area, emerging research suggests that pre-SMA serves higher-order cognitive-linguistic functions such as lexical disambiguation, context-tracking, monitoring, or inhibition of erroneous language representations (27). Furthermore, the pre-SMA and proper-SMA have cytoarchitectural gradients that correspond to functional subregions (57), which further supports the notion that task-fMRI may have the power to identify subregions. Utilizing our within-block segmentation strategy, we were able to discover unique functional sub-regions within pre-SMA that were engaged during the task (see Figure 7 and Table 3). Note that easier stimuli (i.e., segment-1) engages different sub-regions across anterior, medial, and posterior pre-SMA. Each subregion from segment 1 has distinct HRF profiles withGABA+ involvement, and Glx was moderately involved only in mid pre-SMA. The more difficult stimuli (i.e., segment-2) and PSU are more localized to mid pre-SMA. While these preliminary results need further validation, the functional parcellation of pre-SMA allows a plethora of interesting scientific and clinically relevant questions (for example, regional blood flow changes in those sub-regions) that can be addressed in future studies.

### Limitations

This study has several limitations. First, while the sample size suits the scope of a proof-of-principle study, we must be cautionary in interpreting the robustness and generalization our results. For MRS baseline optimization, 14 younger and 14 older participants recruited into this study resulted in significant differences in both GABA+ and Glx between groups. However, when combined with task-fMRI for multimodal fusion, the number of participants was reduced to 9 younger and 10 older participants. After reducing the number of participants, the GABA+ differences between groups were only trending. Based on the effect size computed with 28 participants, a minimum of 22 participants are required to show aging-related differences in GABA+, which is consistent with simulated estimates of sample size for MEGA-PRESS (58). Our multimodal participant numbers were small despite recruiting more than double the reported data. Data from this pilot study combining baseline-MRS and task-fMRI in an aging model was very conservatively promoted. While combining multi-modal datasets is not trivial, future studies should develop better approaches for data fusion keeping a balance between conservative QC criteria while not losing multiple datasets.

Another limitation is that quantifying Glx from difference spectra is suboptimal. Recent studies have shown that Glx quantified from the MEGA-PRESS difference spectra does not conform to the Glx quantified from a short-echo PRESS (59, 60), while other reports identify a significant correlation between PRESS and MEGA-PRESS Glx (61). This is because the glutamate–glutamine complex is inefficiently co-edited during the acquisition tuned to edit GABA. Furthermore, Glx has a relatively short T2, which causes a significant amount of Glx signal to decay away at the optimal GABA+ editing TE of 68 ms. Nevertheless, we did not determine the Glx concentration with “edit off” spectra for three reasons: (1) the expert consensus on j-edited difference spectra analysis has determined that computing non-overlapping resonances can be quantified with spectral fitting using optimized basis sets (62), (2) our dataset lends itself to a good quality fit (Glx CRLB < 4%), which is key to accurate quantification of Glx in the difference spectra (63), and (3) optimizing the off spectrum baseline fit for an aging-cohort was outside of the scope of this report. Future studies in older cohorts should consider acquiring a MEGA-PRESS to collect GABA+ and a short-echo PRESS to collect Glx, if time and scanner resources permit.

It has previously been shown that measuring an individual participant’s macromolecule spectrum can improve baseline fitting and brain metabolite quantification (42, 43), but in this report we did not measure a macromolecule baseline. This may be of particular importance due to the aging-related changes in macromolecule contributions to the MR spectra (18). Perhaps the observation of a double peak in the younger cohort’s 3 ppm GABA moiety compared to the single peak in the older may be due to a lower macromolecule and lipid contamination in younger (Figure 2). Therefore, if time permits, future studies should evaluate the effectiveness of adding a macromolecule baseline measurement in quantifying aging-related differences of neurometabolites.

In our MRS data, only CSF-tissue correction was applied for a conservative approach, but evidence suggests that alpha-based gray matter and white matter tissue correction (34) should be considered. It has been shown that aging-related GABA+ differences are ameliorated after applying alpha-based tissue correction (64, 65). We chose to perform CSF-tissue correction only (corresponding to alpha = 1) because overestimating alpha causes linear deviations from the true value, while underestimating alpha causes non-linear deviations (34). Because it is unknown if alpha remains constant across the lifespan, we chose to overestimate alpha by using CSF-correction as a conservative correction approach. At the very least, the CSF-correction accounts for increased CSF voxel content due to atrophy.

## Conclusion

Considering that semantic fluency is an ergonomic task that is quite appealing to clinical translation such as neurocognitive rehabilitation, examining the interplay between neurovascular and neurochemical underpinnings of such a task, especially in aging, was the main motivation of this study. In the process, we have shown that optimized spline-knot spacing of the baseline in MEGA-PRESS modeling enhances the aging-related GABA+ and Glx differences. We then further promoted the optimally analyzed GABA+ and Glx concentration values to inform language task-fMRI hemodynamics (both standard and neuro-sensitized) in pre-SMA. We note that latency and dispersion parameters from standard BOLD are interesting aging-specific biomarkers for vascular interplay with resting GABA+ and Glx. From a neuroenergetics standpoint, we observe that the neuro-sensitized BOLD requires an (aging-dependent) efficient GABA+ and Glx availability, especially to process metabolically demanding difficult task stimuli. Finally, our multimodal approach also shed light on new discoveries regarding sub-regions within pre-SMA with different hemodynamic characteristics that had unique relationships to underlying GABA+ and Glx.

## Data availability statement

The raw data supporting the conclusions of this article will be made available by the authors, without undue reservation, to any qualified researcher.

## Ethics statement

The studies involving human participants were reviewed and approved by the joint Atlanta VA and Emory University Ethics Board. The patients/participants provided their written informed consent to participate in this study.

## Author contributions

LK and VK: conceptualization of the study, methodological development, MR data collection, subject screening, and manuscript writing. BC: task design. LK, VK, IP, NR, BS, and EA: data analysis. LK, VK, and BC: manuscipt editing. All authors contributed to the article and approved the submitted version.
