# Supplementary material for: The association between language-based task-functional magnetic resonance imaging hemodynamics and baseline GABA+ and glutamate–glutamine measured in pre-supplementary motor area: A pilot study in an aging model
Source: Front Psychiatry. 2022 Aug 15;13:904845. doi: 10.3389/fpsyt.2022.904845 (PMC9421126; doi:10.3389/fpsyt.2022.904845)

Supplementary Section for:

**The association between language-based task-fMRI hemodynamics and baseline GABA+ and glutamate-glutamine measured in pre-SMA: A pilot study in an aging model**

Lisa C. Krishnamurthy^1,2,3^, Isabella Paredes Spir^1^, Natalie O. Rocha^1,4^, Brian J. Soher^5,6^, Edward J. Auerbach^7,8^, Bruce A. Crosson^1,9,10^, Venkatagiri Krishnamurthy^1,10,11^*

^1^Center for Visual and Neurocognitive Rehabilitation, Atlanta VA Healthcare System, Decatur, GA, United States, ^2^Dept. of Physics & Astronomy, Georgia State University, Atlanta, GA, United States, ^3^Dept. of Radiology and Imaging Sciences, Emory University, Atlanta, GA, ^4^Dept. of Biology, Georgia State University, Atlanta, GA, United States, ^5^Department of Radiology, Duke University, Durham, NC, United States, ^6^Center for Brain Imaging and Analysis, Duke University, Durham, NC, United States, ^7^Dept. of Radiology, University of Minnesota, Minneapolis, MN, United States, ^8^Center for Magnetic Resonance Research, University of Minnesota, Minneapolis, MN, United States, ^9^Dept. of Psychology, Georgia State University, Atlanta, GA, United States, ^10^Dept. of Neurology, Emory University, Atlanta, GA, United States, ^11^Dept. of Medicine, Division of Geriatrics and Gerontology, Emory University, Atlanta, GA, United States,

*Supplementary Section1: GABA planning in pre supplementary motor area (preSMA)*

The following steps were taken at the scanner console to plan the MRS voxel on each individual participant’s high resolution T1w image in native space. These instructions were given to trained study personnel to follow at each data acquisition. The instructions are tailored to the Siemens 3T Prisma platform, SYNGO version VE11C.

1. Re-slice T1w image in 3D Tab and save axial and coronal MPR files
2. Open FASTESTMAP and drag resliced T1w image into Exam tab for planning
3. Plan GABA voxel
   1. Find anterior commissure (AC) and posterior commissure (PC) line on re-sliced T1w image
   2. From AC-PC line, find a perpendicular line that extends from AC upwards
   3. Place posterior corner of voxel anterior to the AC line (see figure below)


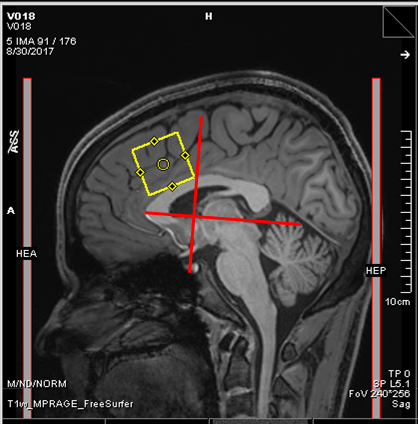


- 1. Rotate voxel parallel to corpus collosum
  2. Move voxel slightly into corpus collosum to avoid the bone
  3. Check the axial and coronal views to ensure midline positioning and that the voxel is not placed over the bone (see figure below)


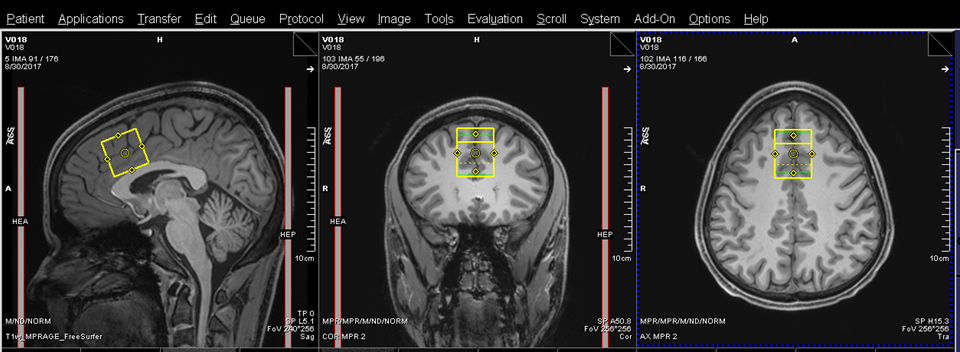


*Supplementary Section 2: LCModel control parameter settings*

Below are the LCModel Control parameter settings used in this study to specify MEGA-PRESS difference spectra fitting. The values deltat, hzpppm, and nunfil are specific to this study (based on the hardware and sequence used). The remaining listed parameters can be applied to other datasets. In this study, we optimized the control parameter dkntmn to 0.6 ppm.

**Supplementary Table 1:** LCModel Control Parameter settings with justification

| **Control Parameter** | **Value** | **Description** |
| --- | --- | --- |
| atth2o | 1.0 | A value of 1.0 removes any default attenuation on the water signal – these can be added back in during postprocessing of signals based on tissue T1 and T2 |
| attmet | 1.0 | A value of 1.0 removes any default attenuation on the metabolite signals – these can be added back in during postprocessing of signals based on metabolite specific T1 and T2 |
| dkntmn | 0.6 | The aging-specific optimized baseline-knot spacing for the MEGA-PRESS difference signal was found to be 0.6 ppm in this study |
| deltat | 0.0005 | The dwell time for this dataset. |
| doecc | T | A value of T (“true”) performs eddy current correction. |
| dows | T | A value of T (“true”) performs water scaling of the metabolite output. |
| hzpppm | 123.258390 | The resonance frequency of the system that this dataset was collected on. |
| nobase | F (T if noBline is used) | A value of F (“false”) indicates that a baseline will be fit and is used in conjunction with the dkntmn value to generate the baseline. A value of T (“true”) indicates that no baseline will be fit. |
| nunfil | 2048 | The number of complex datapoints per FID. |
| ppmend | 0.5 | The minimum frequency in ppm that will be modeled. |
| ppmgap(1,1) | 1.95 | The maximum frequency in ppm that prescribes the gap in the modeled spectrum. A gap is used to avoid areas in the spectra that may have large artifacts. |
| ppmgap(2,1) | 1.2 | The minimum frequency in ppm that prescribes the gap in the modeled spectrum. |
| ppmst | 4.0 | The maximum frequency in ppm that will be modeled. |
| sptype | ‘mega-press-3’ | Informs LCModel that this is a mega-press difference spectrum. |

*Supplementary section 3: Identifying a neurophysiological model to describe HRF metrics and Z(AUC)*

The HRF metrics amplitude, time-to-peak, and FWHM shown in **Figure 5** were further promoted to the model along with neurophysiological parameters GABA, GLX, and Age using 7 models described in **Equations 2-8**. The model that best fit the behavioral data was chosen via the following criteria:

1. The ANOVA of the model must reach significance at p≤0.01(indicated by **) or be trending at 0.011<p<0.05 (indicated by *).
2. Within the significant/trending models, the adjusted R^2^ must be maximum.

The adjusted R^2^ will always be lower than the R^2^, as the adjusted R^2^ takes into consideration the addition of modeling parameters. Due to this feature, the adjusted R^2^ can be compared across models with different number of parameters to identify the most robust model that describes an HRF metric. The **Supplementary Table 2** summarizes the adjusted R^2^ for each model and indicates with Asterix whether the ANOVA reached trending (*) or significance (**). The final chosen model is highlighted in yellow within **Supplementary Table 2**.

**Supplementary Table 2:** Adjusted R^2^ for models that describe HRF amplitude, time-to-peak and FWHM

|  | **Glx only** | **GABA only** | **GABA *Glx** | **Age only** | **Age *Glx** | **Age *GABA** | **Age*Glx *GABA** |
| --- | --- | --- | --- | --- | --- | --- | --- |
| **All participants** | | | | | | | |
| HRF Amplitude | -0.06 | -0.05 | -0.12 | 0 | -0.05 | -0.06 | -0.16 |
| HRF time-to-peak | 0.08 | -0.05 | 0.14 | **0.24*** | 0.21 | 0.24 | 0.13 |
| HRF FWHM | -0.05 | 0.12 | 0.07 | 0.09 | 0.10 | 0.15 | **0.50*** |
| **Younger participants** | | | | | | | |
| HRF Amplitude | -0.01 | -0.08 | -0.04 |  | | | |
| HRF time-to-peak | -0.02 | 0 | -0.25 |  |  |  |  |
| HRF FWHM | -0.14 | -0.14 | -0.49 |  |  |  |  |
| **Older participants** | | | | | | | |
| HRF Amplitude | -0.11 | 0.02 | 0.07 |  | | | |
| HRF time-to-peak | 0.14 | -0.07 | 0.41 |  |  |  |  |
| HRF FWHM | 0.04 | 0.13 | **0.63*** |  |  |  |  |

*ANOVA of model is trending at 0.01<p<0.05

**ANOVA of model is significant at p≤0.01

**Supplementary Table 3:** Adjusted R^2^ of neurophysiological models fit to unsensitized and neurosensitized Z(AUC) for all participants

|  | **Glx only** | **GABA only** | **GABA *Glx** | **Age only** | **Age *Glx** | **Age *GABA** | **Age*Glx *GABA** |
| --- | --- | --- | --- | --- | --- | --- | --- |
| **Unsensitized Z(AUC)** | | | | | | | |
| ROI 1 Z(AUC) | 0.10 | 0.04 | 0 | **0.24*** | 0.15 | 0.20 | 0.11 |
| ROI 2 Z(AUC) | 0.33** | 0.07 | 0.24 | **0.59**** | 0.58* | 0.58** | 0.50* |
| ROI 3 Z(AUC) | 0 | 0.02 | -0.10 | 0 | -0.12 | -0.03 | -0.19 |
| ROI 4 Z(AUC) | 0.07 | 0.03 | 0.03 | **0.19*** | 0.08 | 0.13 | 0 |
| ROI 5 Z(AUC) | 0.05 | -0.05 | -0.02 | **0.18*** | 0.07 | 0.10 | 0.06 |
| $\downarrow$  removal of baseline CBF from task-fMRI Z(AUC)  $\downarrow$ | | | | | | | |
| **Neurosensitized Z(AUC)** | | | | | | | |
| ROI 1 Z(AUC) | 0.02 | 0.01 | -0.09 | 0.15 | 0.05 | 0.12 | 0.05 |
| ROI 2 Z(AUC) | 0.28** | 0.05 | 0.19 | 0.55** | 0.54** | **0.56**** | 0.47* |
| ROI 3 Z(AUC) | **0.24*** | 0.11 | 0.15 | 0.17* | 0.16 | 0.20 | 0.12 |
| ROI 4 Z(AUC) | 0.16* | 0.06 | 0.14 | **0.24*** | 0.16 | 0.22 | 0.11 |
| ROI 5 Z(AUC) | 0.08 | -0.04 | 0.01 | **0.20*** | 0.10 | 0.13 | 0.08 |

*Supplementary Section 4: Relationships from Tables 2 and 3*

The plots below depict the leverage plots for relationships in Table 2 in the main text.


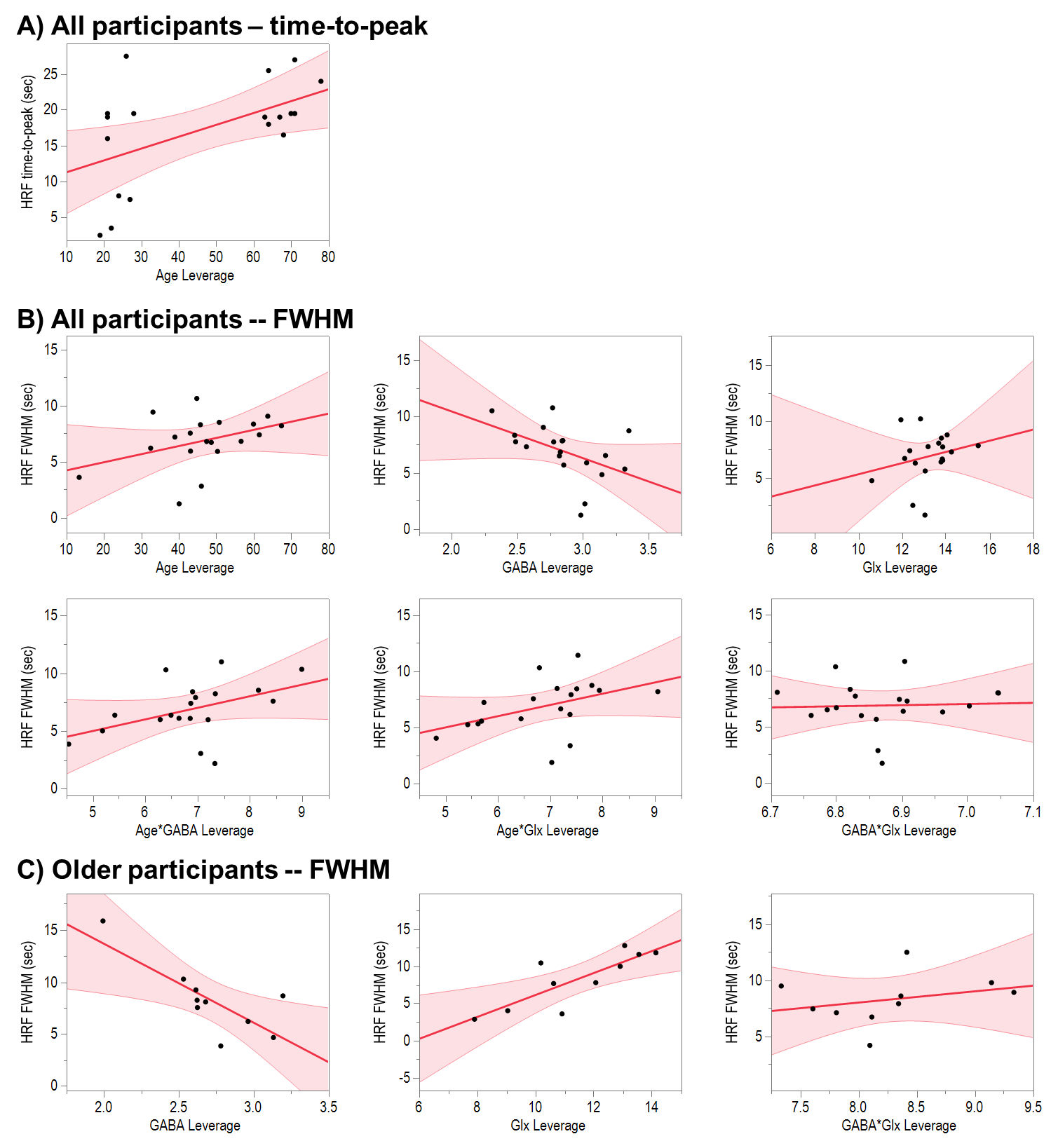


The plots below depict the leverage-by-residual plots for relationships in Table 3 of the main text. The plots are only shown for neuro-sensitized Z(AUC).


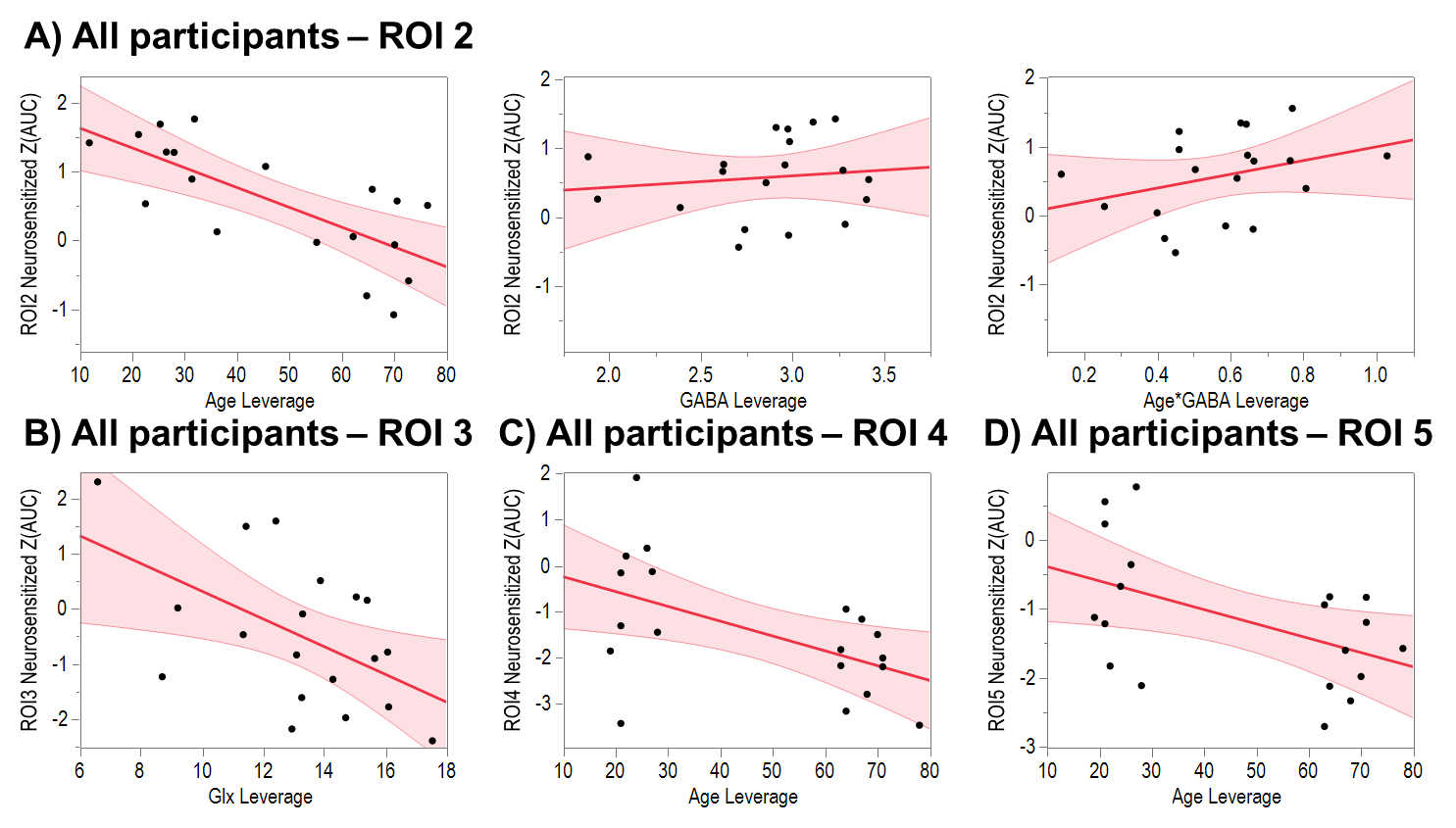

Supplement: Supplementary file 1 [file Data_Sheet_1.DOCX]
